# Dopamine Receptor Genes and Evolutionary Differentiation in the Domestication of Fighting Cocks and Long-Crowing Chickens

**DOI:** 10.1371/journal.pone.0101778

**Published:** 2014-07-31

**Authors:** Tomoyoshi Komiyama, Hisakazu Iwama, Naoki Osada, Yoji Nakamura, Hiroyuki Kobayashi, Yoshio Tateno, Takashi Gojobori

**Affiliations:** 1 Department of Clinical Pharmacology, Tokai University School of Medicine, Shimokasuya, Isehara, Kanagawa, Japan; 2 Kagawa University, Life Science Research Center, Kagawa, Japan; 3 National Institute of Genetics, Shizuoka, Japan; 4 National Research Institute of Fisheries Science, Fisheries Research Agency, Kanagawa, Japan; 5 School of New Biology, Daegu Gyoungbuk Institute of Science and Technology, Daegu, Republic of Korea; 6 SOKENDAI, Department of Genetics, Graduate School of Advanced Studies, Hayama, Kanagawa, Japan; 7 CBRC, BESE, King Abdullah University of Science and Technology, Thuwal, KSA; Natural History Museum of Denmark, Denmark

## Abstract

The chicken domestication process represents a typical model of artificial selection, and gives significant insight into the general understanding of the influence of artificial selection on recognizable phenotypes. Two Japanese domesticated chicken varieties, the fighting cock (*Shamo*) and the long-crowing chicken (*Naganakidori*), have been selectively bred for dramatically different phenotypes. The former has been selected exclusively for aggressiveness and the latter for long crowing with an obedient sitting posture. To understand the particular mechanism behind these genetic changes during domestication, we investigated the degree of genetic differentiation in the aforementioned chickens, focusing on dopamine receptor D2, D3, and D4 genes. We studied other ornamental chickens such as *Chabo* chickens as a reference for comparison. When genetic differentiation was measured by an index of nucleotide differentiation (*N*_ST_) newly devised in this study, we found that the *N_ST_* value of DRD4 for *Shamo* (0.072) was distinctively larger than those of the other genes among the three populations, suggesting that aggressiveness has been selected for in *Shamo* by collecting a variety of single nucleotide polymorphisms. In addition, we found that in DRD4 in *Naganakidori*, there is a deletion variant of one proline at the 24^th^ residue in the repeat of nine prolines of exon 1. We thus conclude that artificial selection has operated on these different kinds of genetic variation in the DRD4 genes of *Shamo* and *Naganakidori* so strongly that the two domesticated varieties have differentiated to obtain their present opposite features in a relatively short period of time.

## Introduction

Domesticated chickens (*Gallus gallus domesticus*) can generally be classified into two categories according to purpose, namely entertainment and consumption. In particular, chickens domesticated for entertainment have attracted people since ancient times with their colorful appearance, masculine posture, and characteristic songs. Lines of evidence also show that chickens were used as religious and political symbols in ancient cultures [1]–[3]. It is therefore easily conceivable that many chicken varieties were established through artificial selection during the domestication process.

In Japan, chickens have also occupied a unique position as edible, ornamental, and religious birds. Japanese people have continued to breed chickens for these purposes for hundreds or possibly thousands of years [4], [5] because these cultural traditions were originally transmitted from mainland China. As a result, there are currently 17 distinct varieties of domesticated chickens known collectively as Japanese ornamental chickens, which exhibit traits not seen in any other chicken varieties worldwide. In particular, many different physical characteristics, such as feather color, tail shape, body size, and comb type, provide us with the unique opportunity to study the effects of artificial selection on those phenotypic traits [6], [7].

In our examination of historical records, we found a description of the fighting cock in the classical Chinese book written in 517 B.C. [8]. This indicates that cockfighting culture must have existed in China over 2500 years before the present day [9]. This is also supported by the mention of long-crowing chickens in the *Liji*, written during the Han period in China (73–49 B.C.) [6], indicating that the long-crowing chicken in China has a history of at least 2000 years. In Japan, the earliest written records of the long-crowing chicken and cockfighting appear in the *Kojiki* (712 A.D.) and *Nihon Shoki* (720 A.D.) [6], [7], [10], which are chronicles of Japan written much more recently than the Chinese records [4]. Thus, these ornamental chickens can serve as an excellent model for studying the relationships of phenotypic and genetic variation under artificial selection for specific human purposes.

In the present study, we examined fighting cocks and long-crowing chickens, two extreme examples of domesticated chickens, to study genetic differentiation affected by artificial selection. The former has been bred for cockfighting with high aggressiveness and the latter for long crowing. Although a variety of cultural aspects toward raising and keeping fighting cocks and long-crowing chickens have been observed not only in Japan but also in other Asian countries such as Thailand, Indonesia, and China, Japanese varieties are known to be maintained in a more rigorous fashion. For example, in Japan, breeding stock of these valuable species have never been interbred with other species; moreover, breeders almost never trade their breeding stock of females with each other [4]. For this reason, we used the Japanese varieties of fighting cocks (*Shamo*) and long-crowing chickens (*Naganakidori*) to study the relationships between human culture and domestication breeding efforts.

The primary goal of this study is to further elucidate the effects of long-term artificial selection among *Shamo* and *Naganakidori*. To accomplish this goal, we performed comparative analyses of the dopamine receptor genes DRD2, DRD3, and DRD4 for three groups of Japanese domesticated chickens: *Shamo*; *Naganakidori*; and one other ornamental chicken, the *Chabo*, as a control. We also analyzed the proline repeat of the DRD4 protein, and predicted the first 3D structures of the receptors of the DRD4 gene.

Dopamine receptors receive dopamine, a catecholamine that has a crucial physiologic role in the regulation of movement, reward, cognition, and emotion [11]. Dopamine is released from presynaptic nerve terminals, where it elicits its effect through a family of G protein-coupled dopamine receptors. These metabotropic G protein-coupled receptors are prominent in the mammalian brain as one of five different subtypes: DRD1, DRD 2, DRD3, DRD4, and DRD5 [11]. The development of molecular cloning techniques subsequently led to the identification of all five dopamine receptors, but physiological classifications as D1-like (DRD1 and DRD5) and D2-like (DRD2, DRD3, and DRD4) still seem practical at present, because many structural, signaling, and pharmacological properties are shared within each subfamily [11]. Moreover, the human dopamine receptors DRD2, DRD3, and DRD4 also have highly similar sequences, suggesting that they are coded for by paralogous genes [12]–[14]. Previous studies have reported on the relationship between dopamine receptor genes and animal behavior. In particular, some dopaminergic neuronal circuits are known to be involved with volition, novelty seeking, and aggression [15]–[28].

Since we intended to study the effects of artificial selection on the two varieties, we devised a new index, *N_ST_*, which can measure the degree of evolutionary differentiation in populations and can also be applied in subdivided populations that differentiated over a relatively short evolutionary time. By applying this new index to the dopamine receptor genes of *Shamo*, we could examine the evolutionary differentiation of specific characteristics in other domesticated chickens. To understand the genetic changes that occurred during domestication for specific purposes, we investigated the degree of genetic differentiation in the effects of artificial selection in these two chicken varieties, specifically focusing on dopamine receptor genes.

## Results

### DRD2, DRD3, and DRD4 Gene Sequences

We sampled blood from eight fighting cocks, nine long-crowing chickens, nine other ornamental chickens, one red junglefowl, and one pheasant. For the 28 samples from the three chicken groups we sequenced DRD2 on chromosome 24 from exon 1 to exon 7 and introns (approximately 3100 bps), DRD3 on chromosome 1 from exon 1 to exon 6 and introns (approximately 2800 bps), and DRD4 on chromosome 5 from exon 1 to exon 4 and introns (approximately 2300 bps). The total number of base pairs sequenced thus was approximately 8200 bps. Therefore, we selected regions surrounding each of 41 sites from the DRD2 gene, 20 sites from the DRD3 gene, and 29 sites from the DRD4 gene, giving a total of 90 segregating sites for analysis (Table S1).

To measure the degree of the evolutionary differentiation among the three domesticated chicken groups, we computed the fixation index (*F_ST_*) [29] for each of the three genes. Population-specific *F_ST_* values were estimated as pairwise *F_ST_* values between focal population samples and the other samples (Table 1). Although the *F_ST_* value of DRD4 for *Shamo* (0.1621) was the highest among the populations, we did not find a clear difference in the values between the three populations. Therefore, we devised *N_ST_*, which can also indicate the evolutionary differentiation between a pair of populations for a nucleotide site as a unit. *N_ST_* is an updated version of *F_ST_* in that we now know that a point mutation occurs at a nucleotide site. In addition, while we need to estimate hetero sites (R, Y, M, K, S, and W) to compute *F_ST_*, we do not need to do it to compute *N_ST_*.

**Table 1:** The population-specific *F_ST_* values of three chicken groups.

Population specific *F_ST_*	Long-crowing (*Naganakidori*)	Fighting cock (*Shamo*)	Ornamental
**DRD2**	0.0314 (P = 0.344)	0.0445 (P = 0.344)	0.0608 (P = 0.318)
**DRD3**	0.0384 (P = 0.334)	0.0453 (P = 0.494)	0.0056 (P = 0.768)
**DRD4**	**0.1379 (P = 0.002)**	**0.1621 (P = 0.002)**	**0.1318 (P = 0.022)**

To explain *N_ST_*, let us first assume that a population is divided into a total of *m* subpopulations, and denote *H_Ti_* as the heterozygosity at the *i*th segregated site of a nucleotide sequence of a total of *n* sites in the population and *H_Sij_* as the heterozygosity of the *i*th segregated site of the sequence in the *j*th subpopulation. Following Nei's *G_ST_*, where *N_STij_* for the *i*-site and the *j*th subpopulation is defined as
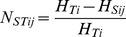
(1)in which
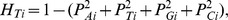
and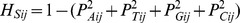,where *P_Ai_*, *P_Ti_*, *P_Gi_*, and *P_Ci_* are the frequencies of the four nucleotides at the *i*th segregated site in the total population, and *p_Aij_*, *p_Tij_*, *p_Gij_*, and *p_Cij_* are those in the *j*th subpopulation. Note that *N_ST_* is defined for each subdivided population.

The *N_STij_* can be used not only for coding regions but also for non-coding regions of genomes in question when nucleotide sites are segregated. If we apply it to introns or non-coding regions in which the evolutionary rate is generally higher than that of exons or RNA coding regions, we can study the evolutionary differentiation for closely related populations, such as the present chicken populations.

We computed *N_STij_* for the individual nucleotide sites in the segregated sites mentioned above, and obtained the mean value, *N_STj_*, for the sites for each of the three chicken populations, as shown below,
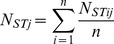
(2)for *j* = 1 to *m*.

We applied *N_ST_* for the three dopamine genes of the domesticated chicken populations to characterize the evolutionary differentiation of the genes in the populations. The results are given in Table 2. It is known that *F_ST_* and *G_ST_* may be negative depending on the allele frequencies in question by using *F_ST_*, which is also true for *N_ST_*. However, the absolute values of the negative values in the present case are so small that we can safely assume they are not significantly different from zero. As a result, the table shows that *N_STi_* of *Shamo* for DRD4 (0.072) is distinctly larger than that of the other groups.

**Table 2:** *N_ST_* for the three dopamine receptor genes among three chicken varieties.

Gene	Species	*N_ST_*
**DRD2**	**Long-crowing (** ***Naganakidori*** **)**	**0.002**
**DRD2**	**Fighting cock (** ***Shamo*** **)**	**−0.001**
**DRD2**	**Ornamental**	**0.001**
**DRD3**	**Long-crowing (** ***Naganakidori*** **)**	**-0.010**
**DRD3**	**Fighting cock (** ***Shamo*** **)**	**0.011**
**DRD3**	**Ornamental**	**0.002**
**DRD4**	**Long-crowing (** ***Naganakidori*** **)**	**−0.022**
**DRD4**	**Fighting cock (** ***Shamo*** **)**	**0.072**
**DRD4**	**Ornamental**	**−0.022**

Values are given per nucleotide site. Negative data values are considered to be zero.

From our results, it is evident that DRD4 is most responsible for the evolutionary differentiation of *Shamo* for greater aggressiveness in fighting.

### 3D model of DRD4 Protein in Various Chicken Varieties

Our analysis demonstrated that there was a significant difference in the mean *N_ST_* values between *Shamo* and the other two groups for the DRD4 gene. A human-rodent comparative study also showed that DRD4 is one of the most rapidly evolving genes in a group of 141 neurotransmitter receptor genes [30]. Here, there are specific polymorphic variants of DRD4 in all of the examined chickens, which is a deletion of one proline at the 24^th^ residue (pro24del) in the nine-proline repeat of exon 1. Our result is in agreement with that of Sugiyama et al. [31] who investigated the proline repeat variation of the DRD4 exon 1 in Phasianidae. Since Phasianidae contains the common ancestor of the chickens studied, we expected the proline repeat to exist in the other chicken groups examined, and aligned their corresponding sequence regions as shown in Table 3. The data show that while the DRD4 proteins of *Shamo*, red junglefowl, and *P. versicolor* have nine proline repeats each, the DRD4 protein of *Naganakidori* (*Koeyoshi* and *Tomaru* breeds) has eight repeats. As three prolines each form a unit in the protein 3D structure, eight repeats is considered to be less advantageous than nine repeats. This structure has three residues per turn and many of the repetitive sequences have a periodicity of three. Other extracellular proline-rich proteins appear to act by binding to proteins on the cell surface and influencing cell-cell recognition [32], [33].

**Table 3:** Alignment of the amino acid sequences corresponding to DRD4 exon 1 for the four Phasianid groups.

Position number of amino acid	1	2	3	4	5	6	7	8	9	10	11	12	13	14	15	16	17	18	19	20	21	22	23	24	25	26	27	28	29	30
Fighting cock (*Shamo*)	M	G	N	G	S	A	G	A	A	P	C	N	G	T	A	P	P	P	P	P	P	P	P	**P**	A	G	H	N	I	A
Long-crowing (*Koeyoshi* and *Tomaru*)	M	G	N	G	S	A	G	A	A	P	C	N	G	T	A	P	P	P	P	P	P	P	P	**-**	A	G	H	N	I	A
Red junglefowl	M	G	N	G	S	A	G	A	A	P	C	N	G	T	A	P	P	P	P	P	P	P	P	**P**	A	G	H	N	I	A
*Phasianus versicolor*	M	G	N	G	S	A	G	A	A	P	C	N	G	T	A	P	P	P	P	P	P	P	P	**P**	A	G	H	N	I	A

The region between the 16^th^ and 24^th^ residues is the proline repeat. In the long-crowing chicken (*Koeyoshi* and *Tomaru*), the proline at the 24^th^ residue is deleted.

**Figure 1:**
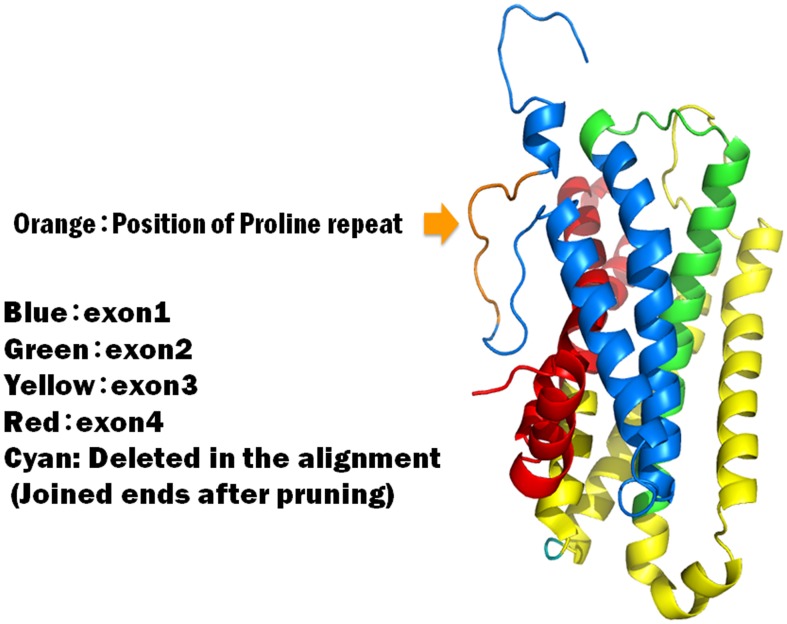
Three-dimensional structure of the DRD4 protein for domesticated chickens. The disordered region (arrow) is the region of proline repeats located on the outside of the cell membrane which receives dopamine released from the synaptic cell.

To confirm the protein interaction, we predicted the 3D structure of DRD4 for the first time by using the MODELLER function of the Insight II program package version 97.2 (BioSym Technologies). As it required a template, we again obtained the crystal structure of the turkey β_1_-adrenergic receptor (PDB code: 2vt4) [34]. The predicted 3D structure shown in Figure 1 illustrates that although the proline repeat was located on an extracellular region, allowing it to react with the ligand, the full exon 3 region was within the intracellular loop of the seven-transmembrane receptor. Therefore, the 3D structure clearly revealed the disadvantageous part in the proline repeat of *Naganakidori*. The irregular structure prevents the chicken DRD4 from properly reacting with the ligand.

## Discussion

The chicken has spread worldwide and differentiated into many varieties to suit specific purposes including food production and entertainment. In this study, we analyzed the evolutionary differentiation of three Japanese domesticated groups, *Shamo*, *Naganakidori*, and other ornamental chickens, because they are typical animals in Japanese culture. The oldest Japanese chronicle, *Chôjû Giga* [6], [7], drawn around 900 years ago and a Japanese national treasure, shows *Shamo* already existing at that time. Those ancient records therefore indicate that Japanese people began breeding chickens to produce their favorite breeds more than 1000 years ago [35].

We investigated difference in selection pressure during artificial selection on the behavior of the chickens in question. For that we chose the three dopamine receptor genes DRD2, DRD3, and DRD4 which are known to be responsible for aggressiveness, among other animal behaviors. Aggressiveness is a very important evolutionary factor for winning mates in order to produce offspring. We then assumed that without knowledge of the mechanics of chicken breeding over the past 1000 years, Japanese people never realized that aggressiveness is adaptive [35]. From this, we examined the evolutionary differentiation of the dopamine genes among the three groups of the domesticated chickens. The problem, however, is that the period of artificial selection is relatively short in the context of evolution, so we might not have been able to detect the degree of evolutionary differentiation among the chickens in question. To solve this problem, we extended *G_ST_* [36] to the nucleotide site level and devised a new index, the nucleotide population diversity, *N_STij_*. As mentioned above, even if we could not measure the degree of population diversity at the gene level, we would be able to measure it at the nucleotide site level in introns or non-coding regions. This proved true for the three dopamine receptor genes, as shown in Table S1, and Tables 1, 2. Table 2 indicates that the *N_STi_* value of DRD4 for *Shamo* is distinctively larger than that of the others, demonstrating that it has been the most differentiated in evolution among the three receptor genes studied. It is possible that artificial selection has operated most heavily on DRD4 among the three populations, indicating that positive selection has diversified DRD4 more than in the other breeds. Table 2 also shows that the *N_STi_* of *Shamo* for DRD4 (0.072) is distinctly larger than that for the other breeds. We took this to imply that DRD4 has a more prominent role than DRD2 and DRD3 in controlling the behavior in chickens. From these results in fighting cocks, artificial increases in aggression might have necessarily led to DNA variations. While there are several genes related with aggressiveness in the animals, we suggest that one of the possible ones is the DRD4 gene examined in this analysis.

Our ancestors utilized artificial selection to produce increasingly stronger fighting cocks through repeated breeding without knowing the mechanics of the process. In the present study, the *N_STij_* values of DRD4 for *Shamo* were highest among the three groups; therefore, we can now confirm that the genetic basis for this behavior, particularly the aggressive fighting disposition that is essential for *Shamo* to attract mates, is in fact the DRD4 gene, although we cannot completely rule out DRD2 or DRD3. This finding suggests that ancient Japanese people performed more artificial selection on fighting cocks than they did on the other breeds of chicken, perhaps because cockfighting was more appealing as entertainment in those days.

*Naganakidori* (*Koeyoshi* and *Tomaru* breeds) might have been subject to selection according to different proline repeats. Crowing may be controlled more directly by genes other than dopamine receptor genes, because the long-crowing chicken has a deficient 3D structure of DRD4. Our prediction of the 3D structure of DRD4 revealed that a lack of proline repeats in an extracellular region made it less effective at reacting with its ligand, enabling *Naganakidori* to keep still and quiet except during long crowing.

**Figure 2:**
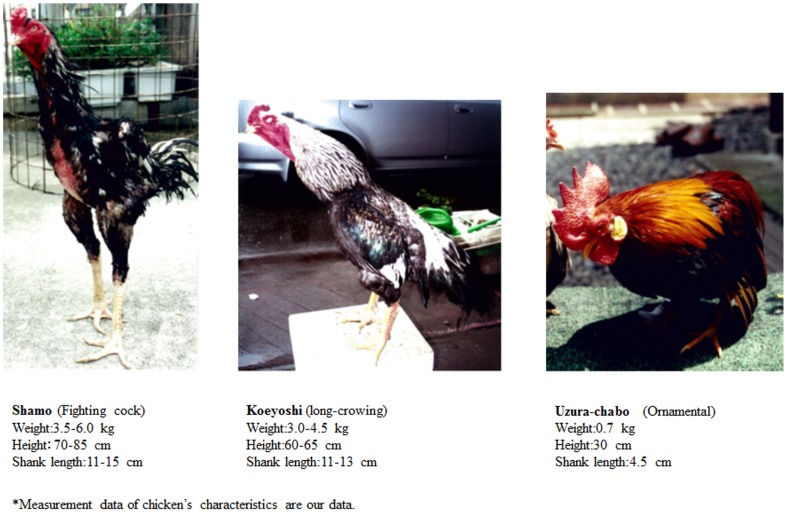
The three domesticated varieties examined in this study. In Japan, there are 17 distinct varieties of ornamental domesticated chickens, which are famous for their characteristic varied body color and forms. These chickens continue to be bred for these different characteristics by breeders.

The lifespan of one *Naganakidori* breed, *Koeyoshi* (Fig. 2), is known to be shorter than that of other varieties. On the other hand, *Koeyoshi* acquired this trait of long crowing through artificial selection. Japanese people long ago must have appreciated the chicken's long crowing duration, and artificial selection was continuously practiced to produce chickens that crowed for increasingly longer. As a result, some *Naganakidori* can now crow for over 15 s [4]. It is noteworthy that *N_ST_* enables us to study the evolutionary differentiation among populations taking place within a relatively short period, and that Japanese domesticated chickens provide us with an excellent model to study how selection works on particular genes. The protein products of genes are certainly involved in the network responsible for animal behavior, and also in aging. The polymorphism of DRD4 in humans is known to link with neurological and psychiatric conditions such as schizophrenia, Parkinson's disease, addictive behaviors, eating disorder, and longevity [37]–[41]. Therefore, further investigation into the aforementioned network will lead us to a deeper understanding of the evolution and pathology of animals.

In this study, the genetic differentiation of *Shamo* from ornamental chickens was found to be its higher DRD genes, which likely serve as the basis for its more aggressive fighting temperament.

## Conclusions

We invented an index, *Nst*, to measure evolutionary differentiation by artificial selection on the three dopamine receptor genes, DRD2, DRD3 and DRD4 for three chicken populations; *Shamo*, *Naganakidori* and *Ornamental* chickens. We draw the conclusion that artificial selection had operated most strongly on DRD4 of *Shamo*. The reason for that, we consider, is that *Shamo* had been used for fighting and artificially selected for more and more aggressive ones. In addition, we determined the 3D structure of DRD4, and found that a *Naganakidori* breed, *Koeyoshi* had a deficient 3D structure that might be the reason why it has a shorter lifespan than other breeds.

Our future research will be conducted in light of these results. We have started to investigate the levels of dopamine, adrenaline, noradrenaline, and other substances in *Shamo* and other chicken populations' layer brains. In addition, we plan to analyze the exomes of ornamental chickens, *Naganakidori*, and *Shamo*. We will aim to find the specific relationship between chicken domestication and its genetic effects.

## Materials and Methods

### Ethics statement

We carried out use of all legitimate samples for our researches. Also, all chicken experimental procedures were carried out in accord with the Fundamental Guidelines for Proper Conduct of Animal Experimentation and Related Activities in Academic Research Institutions, under the jurisdiction of the Ministry of Education, Culture, Sports, Science and Technology in Japan, and reviewed and approved by The Institutional Animal Care and Use Committee at the National Institute of Genetics.

### Sample Collection

The varieties of chicken examined in this study are given in Table 4 and Figure 2. Dopamine receptor D2, D3, and D4 genes (DRD2, DRD3, and DRD4) were cloned from the blood of domesticated chicken. We obtained a total of 28 samples, fighting cocks (*Shamo*; eight samples), long-crowing chickens (*Naganakidori*; nine samples), other ornamental chickens (nine samples), and one red junglefowl (*G. gallus*), from the Bird Center of Kurume in Fukuoka prefecture. We also took samples from a pheasant (*Phasianus versicolor*) from the Nogeyama Zoological Gardens of Yokohama in Kanagawa prefecture. These domesticated chickens had been collected in our previous studies [4], [5], [35].

**Table 4:** List of samples from eight fighting cocks (*Shamo*), nine long-crowing chickens (*Naganakidori*), nine ornamental chickens, one red junglefowl, and one pheasant.

English Name	Sample No. & Japanese Name	DRD2 Accession No.	DRD3 Accession No.	DRD4 Accession No.	Sex	Location
Fighting cock	49 Shamo	AB688642	AB692966	AB699005	Male	Japan: Ibaraki
Fighting cock	98 Shamo	AB688643	AB692967	AB699006	Female	Japan: Okinawa
Fighting cock	100 Shamo	AB688644	AB692968	AB699007	Male	Japan: Tochigi
Fighting cock	102 Shamo	AB688645	AB692969	AB699008	Female	Japan: Chiba
Fighting cock	112 Shamo	AB688646	AB692970	AB699009	Male	Japan: Okinawa
Fighting cock	113 Shamo	AB688647	AB692971	AB699010	Male	Japan: Okinawa
Fighting cock	126 Shamo	AB688648	AB692972	AB699011	Female	Japan: Okinawa, Ishigaki
Fighting cock	149 Shamo	AB688649	AB692973	AB699012	Male	Japan: Nagasaki, Tsushima
Long-crowing chicken	27 Koeyoshi	AB688650	AB692974	AB699013	Male	Japan: Iwate
Long-crowing chicken	28 Koeyoshi	AB688651	AB692975	AB699014	Male	Japan: Aomori
Long-crowing chicken	29 Koeyoshi	AB688632	AB692976	AB699015	Female	Japan: Aomori
Long-crowing chicken	30 Koeyoshi	AB688652	AB692977	AB699016	Female	Japan: Iwate
Long-crowing chicken	56 Totenko	AB688653	AB692978	AB699017	Male	Japan: Kanagawa
Long-crowing chicken	57 Totenko	AB688654	AB692979	AB699018	Female	Japan: Kanagawa
Long-crowing chicken	203 Tomaru	AB688655	AB692980	AB699019	Male	Japan: Niigata
Long-crowing chicken	204 Tomaru	AB688656	AB692981	AB699020	Female	Japan: Niigata
Long-crowing chicken	205 Tomaru	AB688657	AB692982	AB699021	Female	Japan: Niigata
Ornamental chicken	6 Kawachi-yakko	AB688634	AB692983	AB699022	Male	Japan: Shizuoka
Ornamental chicken	13 Koshamo	AB688630	AB692984	AB699023	Male	Japan: Shizuoka
Ornamental chicken	16 Katsura-chabo	AB688631	AB692985	AB699024	Male	Japan: Shizuoka
Ornamental chicken	20 Katsura-chabo	AB688658	AB692986	AB699025	Female	Japan: Shizuoka
Ornamental chicken	35 Satsumadori	AB688633	AB692988	AB699027	Male	Japan: Kagoshima
Ornamental chicken	36 Uzura-chabo	AB688636	AB692989	AB699028	Male	Japan: Ibaraki
Ornamental chicken	42 Nankin-shamo	AB688637	AB692990	AB699029	Male	Japan: Ibaraki
Ornamental chicken	59 Shokoku	AB688638	AB692991	AB699030	Female	Japan: Shizuoka
Ornamental chicken	90 Minohikidori	AB688639	AB692992	AB699031	Male	Japan: Shizuoka
Red jungle fowl	222Sekishoku yakei	AB688659	AB692995	AB699034	Male	Japan: Fukuoka
Pheasant (*Phasianus versicolor)*	207 Nihonkiji	AB688660	AB692996	AB699035	Male	Japan: Kanagawa

### DNA Extraction, PCR Amplification, Sequencing, and Cloning

Blood was taken from each chicken, and each sample was suspended in 400 µl of TNES-8M urea. Twenty microliters of proteinase K (20 mg/ml) and 1M dithiothreitol (DDT) were added to the samples, which were then incubated for 1–5 h at 60°C. Each incubated sample was subsequently mixed with 500 µl of a phenol/chloroform/isoamyl alcohol solution (25∶24∶1) for 3 min; this was repeated twice. After precipitation with 2–2.5 vol. of ethanol, the pellets were rinsed in 70% cold ethanol and dried. The samples were then dissolved in TE buffer [10 mM Tris–HCl (pH 8.0), 1 mM EDTA]. Regions of the dopamine receptor genes were amplified using the polymerase chain reaction (PCR), the primers for which are given in Table 5. PCR was used to amplify dopamine receptor D2, D3, and D4 genes. The PCR enzymes used were KOD-Plus and KOD-Plus (TOYOBO, Osaka, Japan) Ver. 2. PCR was performed under the following conditions: denaturation for 2 min at 94°C; 30–33 cycles of 94°C for 15–30 s, annealing at 50–57°C for 15–20 s, and extending at 68°C for 1 min/kb. The PCR products were purified using MinElute PCR Purification Kit (QIAGEN, Duesseldorf, Germany) and ExoSAP-IT (USB Corp, Ohio, America), and were then directly sequenced using Big Dye Terminator Chemistry Ver.3.1 and an ABI Prism 3730xl DNA sequencer (ABI, California, America). We also cloned exon 1 of the DRD4 gene to confirm the sequence using independently amplified PCR products. A PCR product of the expected size (1046 bp) was amplified with primers F6456 and R7502. The band corresponding to the PCR product was excised from a 1.5% agarose gel (Bio-Rad, California, America). After purification with the MinElute Gel Extraction Kit (QIAGEN), the DNAs were ligated into the TaKaRa T-Vector pMD20 using Takara DNA Ligation kit <Mighty Mix>. The products were used to transform *Escherichia coli* (DH5α). Transformants were plated onto LB-agar plates containing the appropriate amounts of ampicillin, X-gal, and IPTG. White recombinant bacteria were grown in 2×LB broth containing ampicillin (100 µg/ml) and the plasmid DNAs were purified using a Montage Plasmid Miniprep Kit (Millipore, Massachusetts, America). The plasmid DNAs were used as templates and directly cycle-sequenced using M13 (-20) f and M13r primers and a Big Dye Terminator Ver.3.1 Cycle Sequencing Kit (ABI). Last, nucleotide sequences were analyzed on an ABI 3730xl automated DNA sequencer.

**Table 5:** Primers used for amplifying the DRD2, DRD3, and DRD4 genes.

Gene name		Forward Primer name	Forward Primer	Reverse Primer name	Reverse Primer
DRD2	Exon1	F01F	5′CAGGTACCTGACCATCAGCTGT	R02R	5′TCCAGTGATGATGACTCCACCAC
DRD2	Exon1	F01F02	5′CAGCTGTGGTCTGGATGTCCG	-	-
DRD2	Exon1	F01F3	5′CAGAAATACGGGGGAATCAA	R02R3	5′CATCCAGCCTTTCCTCAGAC
DRD2	Exon2-5	F03F	5′ATCCTGCTTCCCTGTTACTGAT	R07R	5′CTGATCTTTAGGTGAGCTTAGTC
DRD2	Exon2-5	F04F	5′GTGCTCCTTCCACTCTGGGCGC	R05R	5′CAGAGGGCGCCCAGAGTGGAAG
DRD2	Exon2-5	F06F	5′TCTGCTTCATGCCCTGTGCTTG	R06R	5′CAAGCACAGGGCATGAAGCAGA
DRD2	Exon2-5	-	-	R15R	5′GAGATTGAATGGTAACCTTTG
DRD2	Exon6-7	F08F	5′TCAGCATCATCCAGCTTCACTC	R9R	5′GTCGAATATTCAGACTGCGTG
DRD2	Exon6-7	F10F	5′ACTCAGGATATAGACAGAGACAT	R09R2	5′GCTGTTGCCTGTCTTGA
DRD2	Exon6-7	-	-	R09R3	5′CATTCAGACTGGGTGCC
DRD2	Exon6-7	-	-	R11R	5′TCATGCCCAGCGCAAGAGCTCG
DRD3	Exon1	F01F	5′GTACATCTACACCAATGCACAT	R01R	5′CAGTCTATAACAGACTTCCACC
DRD3	Exon1	F16	5′CAGGAGGAGCTAGAA	R180	5′GACTTGATATCATCATT
DRD3	Exon1	F42	5′GGAGCAAAACTATGACTT	R224	5′ATGTCAGCCTTGTTG
DRD3	Exon2	F02F	5′CACTCAGGATACTTCTCCAACA	R02R	5′TCATCGCAACCCAAAGCGCTATC
DRD3	Exon2	F02F2	5′ACTTCTCCAACACCACCCTG	R02R2	5′CCCAAAGCGCTATCATATCC
DRD3	Exon3	F03F	5′GACTTGAAGTAGCATCGCAAAT	R03R	5′CTGGCCTATCCTGAATGCACAT
DRD3	Exon4	F01	5′ATTCATCTTGATGTTGAGG	-	-
DRD3	Exon4	F04F	5′CTGAGCATCACAGTGAACACAA	R04R	5′GCTTATGTTCCTGAGGATGCT
DRD3	Exon5	F05F	5′ATCTCTTCATCACTTCTACCAT	R05R	5′CATATAAAGAGCAGCTTCACTG
DRD3	Exon5	-	-	R243	5′TTGTTCAGGATGTCACTTAC
DRD3	Exon6	F06F	5′ATCCAGCATATTACCTGCTTTGC	R06R	5′TGCTTTCCATTCTGTGCTTTGA
DRD3	Exon6	-	-	R06R2	5′TGCTTTCCATTCTGTGCTTTG
DRD3	Exon7and 8	F0708F	5′ACAGAGGCTCAGTAATGGCAAA	R0708R	5′CAGGTACTGCAGCAGTCAGGAT
DRD4	Exon1	F06	5′CGCAGCGCTTGTGGGACCG	R494	5′GCTCCGTGCACACGCTGAGGCA
DRD4	Exon1	F190	5′ATCCCGGTGAGCGCGTTACTG	R679	5′GTCCAGGATGCCAGCAGG
DRD4	Exon1	F494	5′TGCCTCAGCGTGTGCACGGAGC	R803	5′TTGAGGAGCTTGGGGCAT
DRD4	Exon1	F6456	5′GATCCACCGCTGTATTCGCAG	R7502	5′TGCTCACAGTGCGACGGCTCCCTG
DRD4	Exon1	F6647	5′GCATCCCGGTGAGCCGCTTACT	R7321	5′CCAGTGGTCCAGGATGCCAGCA
DRD4	Exon1	F7126	5′ CCTGCTGGCATCCTGG 3′	R6770	5′GCATGGAGCCGCCGAGC 3′
DRD4	Exon2	F6089	5′TCAGTCCTGGTACCCAGCACA	R6514	5′GCCGGTTGTAGTTCAGTGGGACT
DRD4	Exon2	F6999	5′GATGCTCCCAGAGGCA	R7349	5′GCCGGTTGTAGTTCAGT
DRD4	Exon3	F21	5′CCTACCTTACCAGTGGTGCTA	R508	5′AGACAGTCTGGAGCCCATTGCT
DRD4	Exon3	-	-	R675	5′CCTGTAGGACCACACAGCAC
DRD4	Exon3	L26	5′ATCAAGCTGCTTCCATTCGCTTAC	H455	5′CTCCGGTTCCATGCCGACCCGC
DRD4	Exon3	L249	5′AGCTTGTGCCAGCTGGAGGATGAC	H730	5′CACTCTCTGGACCGGGTTCAAC
DRD4	Exon4	F7337	5′GTCACGGAGAGATGGTAGCTCTGCA	R7740	5′CATCCTGGAGCTGCCATCCTGG
DRD4	Exon4	F2	5′GCTTTCCTCTTCTGCTGGACA	R3	5′CAAGACTTTGCGGAAGAA
DRD4	Exon4	F22	5′AACTCCAACCCTTTTGAACACA	R331	5′ACCTTAGGCAGAAATAGATC

### Sequence Alignment

The 28 nucleotide sequences surrounding each of DRD2, DRD3, and DRD4 were aligned using CLUSTAL W [42] to compute *N_S_*_T_ among the three chicken groups. After excluding gaps the total number of nucleotides for each sequence was 8200 bp.

### *F_ST_* analyses of DRD2, DRD3, and DRD4 Gene Sequences

DNA haplotypes were estimated using PHASE (University of Chicago, Chicago, IL) [43] as implemented in DnaSP v5 software (Universitat de Barcelona, Barcelona, Spain) [44]. Wright's *F_ST_* statistics were estimated by the method of Hudson et al. using in-house Perl script [45] and statistical significance was evaluated using the permutation test with 1000 replications.

### Protein Analysis

We predicted the 3D structure of the chicken DRD4 protein by using the homology modeling software, MODELLER [46] function of the Insight II program package version 97.2 (BioSym Technologies, San Diego, CA). In this study, we used the crystal structure of the turkey β_1_-adrenergic receptor (PDB code: 2vt4) as a template [34] because this sequence is highly similar to that of DRD4.

## Supporting Information

Table S1 **List of 90 bp segregation sites of the DRD2, DRD3, and DRD4 genes in each of the three domesticated varieties.** The position of segregation sites were referenced No. GCA_000002315.2 into the Galgal 4 database of Ensembl. R:G or A, Y:T or C, M:A or C, K:G or T, S:G or C, W:A or T.
(XLS)
